# 6-{5-Amino-3-*tert*-butyl-4-[(*E*)-(3-methyl-1,2,4-thiadiazol-5-yl)diazen­yl]-1*H*-pyrazol-1-yl}-1,3,5-triazine-2,4(1*H*,3*H*)-dione–1-methyl­pyrrolidin-2-one–water (1/1/1)

**DOI:** 10.1107/S1600536810010871

**Published:** 2010-03-27

**Authors:** Hiroki Shibata, Jin Mizuguchi

**Affiliations:** a Department of Applied Physics, Graduate School of Engineering, Yokohama National University, Tokiwadai 79-5, Hodogaya-ku, Yokohama 240-8501, Japan

## Abstract

In the title compound, C_13_H_16_N_10_O_2_S·C_5_H_9_NO·H_2_O, the entire 1-methylpyrrolidin-2-one (NMP) mol­ecule is disordered over two sites with occupancies of 0.488 (5) and 0.512 (5). The six-membered triazine ring and the two five-membered pyrazole and thiadia­zole rings, together with the diazene (–N=N–) linkage are almost coplanar (r.m.s. deviation for the non-H atoms = 0.0256 Å) with methyl groups from the *tert*-butyl substituent on the pyrazole ring located above and below the plane. Three intra­molecular N—H⋯N hydrogen bonds contribute to the planarity of the system. The O atom of the NMP mol­ecule is hydrogen bonded to an O—H group of water. In turn, the water mol­ecule is hydrogen bonded to the mono-azo skeleton through inter­molecular N—H⋯O and O—H⋯N hydrogen bonds. At both ends of the long mol­ecular axis of the main mol­ecule there are inter­molecular N—H⋯N hydrogen bonds, arranged in a head-to-tail fashion, between the N—H group of the triazine ring of one mol­ecule and the N atom of the thia­diazole ring of a neighboring mol­ecule. These form a polymeric chain along [110] or [1
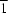
0]. The main mol­ecules are stacked alternately along the *b* axis, which effectively cancels their dipole moments. In addition, pairs of alternate molecules are dimerized *via* inter­molecular hydrogen bonds involving the solvent mol­ecules.

## Related literature

For details of azo pigments, see: Herbst & Hunger (2004 ▶). For the structure of the Na(I) complex of the related bis-azo compound, see: Shibata & Mizuguchi (2010 ▶). For the synthesis of the title compound, see: Nagata & Tateishi (2009 ▶). 
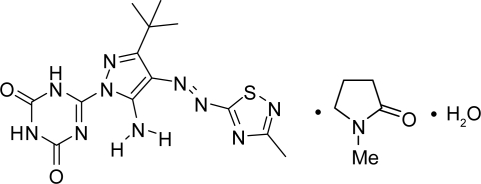


## Experimental

### 

#### Crystal data

C_13_H_16_N_10_O_2_S·C_5_H_9_NO·H_2_O
*M* *_r_* = 493.54
Monoclinic, 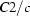

*a* = 27.8283 (5) Å
*b* = 7.0269 (1) Å
*c* = 23.4417 (4) Å
β = 91.3430 (7)°
*V* = 4582.69 (13) Å^3^
*Z* = 8
Cu *K*α radiation
μ = 1.70 mm^−1^
*T* = 93 K
0.50 × 0.10 × 0.10 mm

#### Data collection

Rigaku R-AXIS RAPID diffractometer
Absorption correction: multi-scan (*ABSCOR*; Higashi, 1995 ▶) *T*_min_ = 0.529, *T*_max_ = 0.844
3977 measured reflections
3977 independent reflections
3083 reflections with *F* ^2^ > 2σ(*F* ^2^)

#### Refinement

*R*[*F* ^2^ > 2σ(*F* ^2^)] = 0.049
*wR*(*F* ^2^) = 0.150
*S* = 1.13
3977 reflections
347 parameters
84 restraints
H-atom parameters constrained
Δρ_max_ = 0.39 e Å^−3^
Δρ_min_ = −0.35 e Å^−3^

### 

Data collection: *PROCESS-AUTO* (Rigaku, 1998 ▶); cell refinement: *PROCESS-AUTO*; data reduction: *CrystalStructure* (Rigaku/MSC & Rigaku, 2006 ▶); program(s) used to solve structure: *SIR2004* (Burla *et al.*, 2005 ▶); program(s) used to refine structure: *SHELXL97* (Sheldrick, 2008 ▶); molecular graphics: *ORTEPIII* (Burnett & Johnson, 1996 ▶); software used to prepare material for publication: *CrystalStructure* (Rigaku/MSC & Rigaku, 2006 ▶).

## Supplementary Material

Crystal structure: contains datablocks global, I. DOI: 10.1107/S1600536810010871/sj2752sup1.cif

Structure factors: contains datablocks I. DOI: 10.1107/S1600536810010871/sj2752Isup2.hkl

Additional supplementary materials:  crystallographic information; 3D view; checkCIF report

## Figures and Tables

**Table 1:** Hydrogen-bond geometry (Å, °)

*D*—H⋯*A*	*D*—H	H⋯*A*	*D*⋯*A*	*D*—H⋯*A*
O4—H4*A*⋯O3*A*	0.90	2.19	3.087 (4)	176
O4—H4*A*⋯O3*B*	0.90	1.87	2.752 (4)	166
O4—H4*B*⋯N7	0.90	2.70	3.104 (2)	109
O4—H4*B*⋯N8	0.90	2.25	3.100 (2)	159
N1—H1*N*⋯N9^i^	0.88	2.07	2.947 (2)	176
N2—H2⋯N5	0.88	2.27	2.654 (2)	106
N2—H2⋯O3*A*^ii^	0.88	1.95	2.778 (4)	157
N2—H2⋯O3*B*^ii^	0.88	2.01	2.766 (4)	143
N10—H10*N*⋯N3	0.88	2.11	2.727 (2)	126
N10—H10*M*⋯O4	0.88	2.19	3.002 (2)	154
N10—H10*M*⋯N7	0.88	2.28	2.804 (2)	119

Symmetry codes: (i) 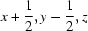; (ii) 
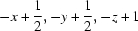
.

**Fig. 1.**
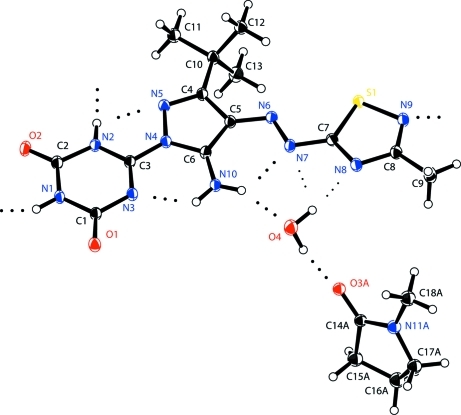
The asymmetric unit of I. Hydrogen atoms except for those involved in hydrogen bonds (dotted lines) are omitted for clarity.

**Fig. 2.**
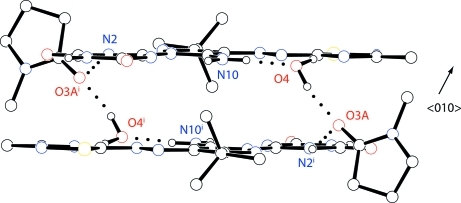
Molecular stack along the <010> axis. Symmetry code: (-x+1/2, -y+1/2, -z+1). Hydrogen bonds are drawn as dotted lines.

## supplementary crystallographic information

### Comment

Azo pigments play an important role as colorants in the imaging and printing industries (Herbst & Hunger, 2004). Compound (I), C_13_H_16_N_9_O_2_S^.^C_5_H_7_NO^.^H_2_O, is a monoazo pigment solvated with an *N*-methyl-2-pyrrolidone (NMP) molecule and water. The background of the present study is set out in our paper on the Na(I) complex with the closely related bis-azo compound as a ligand (Shibata & Mizuguchi, 2010). We report here on the structure of a Na^+^-free monoazo compound isolated from the same reaction mixture that produced the Na(I) complex.

Fig. 1 shows the *ORTEP* plot of I. The six-membered C1–C3,N1–N3 and two five-membered C4–C6,N4,N5 and S1,C7,C8,N8,N9 rings together with the N6–N7 azo linkage lie in a plane (rms deviation for the non-H atoms 0.0256Å) with a methyl group from the *t*-butyl substituent on the pyrazol ring above and below that plane. The formation of three intramolecular hydrogen bonds: N2—H2···N5, N10—H10M···N7, and N10—H10N···N3, Table 1, stabilises this planar conformation. The O4 atom of the water molecule is nearly on the same plane of the monoazo molecule: the dihedral angle between the planes N10/C6/H10M/H10M and O4/N10/H10M/H10N: 1.0 (1)°. However, the best fit planes through the NMP solvent molecule (C14A—C17A/N11A/O5A) and that of the water molecule (H4A/O4/H4B) are inclined to the above bis-azo skeleton by 124.6 (0) and 113.7 (0)°, respectively. The water molecules are hydrogen bonded to the O3A or O3B atoms of the disordered NMP molecule though O4—H4A···O3 hydrogen bonds. In turn, the O4 atom is hydrogen-bonded to the H10M—N10 amino group of the monoazo skeleton. In addition, the O4—H4B group is weakly hydrogen-bonded to both N7 and N8. At both ends of the long molecular axis of the main molecule, there are intermolecular N1—H1···N9 hydrogen bonds. These form a one-dimensional polymer chain on the molecular plane along the long molecular axis: <110> or <1-10> direction.

As shown in Fig. 2, the monoazo molecules are alternately stacked along the <010> direction in such a way to cancel their dipole moments so as to electrostatically stabilize themselves in the crystal. Each alternating pair is linked through a set of three-consecutive intermolecular hydrogen bonds. On one side of the molecule: N2—H2 (triazine ring)···O3A^i^ or O3B^i^ (NMP), O3A^i^ or O3B^i^ (NMP)···H4A^i^—O4^i^ (water), and O4^i^(water)···H10M^i^—N10^i^ (amino group) [symmetry code: (i) (-x+1/2, -y+1/2, -z+1)]. An equivalent set of H-bonding interactions are found at the opposite sides of the molecules.

### Experimental

The title compound was synthesized as described by Nagata *et al.* (2009). The structure reported here is of the Na(I) cation free product which made up approximately 20% of the product mixture by emission spectrochemical analysis. A single crystal suitable for X-ray analysis was grown from a solution in *N*-methy-2-pyrrolidone prepared at 100 °C. Needle shaped crystals were obtained after standing for one week.

### Refinement

The entire NMP molecule was disordered over two sites (C14A—C18A/N11A/O3A and C14B—C18B/N11B/O3B) with occupancies of 0.488 (5) and 0.512 (5), respectively. These non-H atoms were refined anisotropically. The occupancies extend to the associated H atoms. All H atoms were placed in geometrically idealized position and constrained to ride on their parent atoms, with C—H in CH_2_ = 0.99, and C—H in CH_3_ = 0.98 Å, and *U*_iso_(H) = 1.2 and 1.5 *U*_eq_(C), respectively, and with O—H = 0.84, and N—H = 0.88 Å and *U*_iso_(H) = 1.2. The low theta fraction is due to a weakly diffracting crystal.

### Crystal data

**Table:**

C_13_H_16_N_10_O_2_S·C_5_H_9_NO·H_2_O	*F*(000) = 2080.00
*M**_r_* = 493.54	*D*_x_ = 1.431 Mg m^−^^3^
Monoclinic, *C*2/*c*	Cu *K*α radiation, λ = 1.54187 Å
Hall symbol: -C 2yc	Cell parameters from 23604 reflections
*a* = 27.8283 (5) Å	θ = 3.2–68.2°
*b* = 7.0269 (1) Å	µ = 1.70 mm^−^^1^
*c* = 23.4417 (4) Å	*T* = 93 K
β = 91.3430 (7)°	Needle, yellow
*V* = 4582.69 (13) Å^3^	0.50 × 0.10 × 0.10 mm
*Z* = 8	

### Data collection

**Table:**

Rigaku R-AXIS RAPID diffractometer	3083 reflections with *F*^2^ > 2σ(*F*^2^)
Detector resolution: 10.00 pixels mm^-1^	*R*_int_ = 0.0000
ω scans	θ_max_ = 68.2°
Absorption correction: multi-scan (*ABSCOR*; Higashi, 1995)	*h* = 0→33
*T*_min_ = 0.529, *T*_max_ = 0.844	*k* = 0→7
3977 measured reflections	*l* = −28→28
3977 independent reflections	

### Refinement

**Table:**

Refinement on *F*^2^	84 restraints
*R*[*F*^2^ > 2σ(*F*^2^)] = 0.049	H-atom parameters constrained
*wR*(*F*^2^) = 0.150	*w* = 1/[σ^2^(*F*_o_^2^) + (0.0944*P*)^2^] where *P* = (*F*_o_^2^ + 2*F*_c_^2^)/3
*S* = 1.13	(Δ/σ)_max_ < 0.001
3977 reflections	Δρ_max_ = 0.39 e Å^−^^3^
347 parameters	Δρ_min_ = −0.35 e Å^−^^3^

### Special details

**Table:**

Geometry. All esds (except the esd in the dihedral angle between two l.s. planes) are estimated using the full covariance matrix. The cell esds are taken into account individually in the estimation of esds in distances, angles and torsion angles; correlations between esds in cell parameters are only used when they are defined by crystal symmetry. An approximate (isotropic) treatment of cell esds is used for estimating esds involving l.s. planes.
Refinement. Refinement of *F*^2^ against ALL reflections. The weighted *R*-factor wR and goodness of fit *S* are based on *F*^2^, conventional *R*-factors *R* are based on *F*, with *F* set to zero for negative *F*^2^. The threshold expression of *F*^2^ > σ(*F*^2^) is used only for calculating *R*-factors(gt) etc. and is not relevant to the choice of reflections for refinement. *R*-factors based on *F*^2^ are statistically about twice as large as those based on *F*, and *R*- factors based on ALL data will be even larger.

### Fractional atomic coordinates and isotropic or equivalent isotropic displacement parameters (Å^2^)

**Table:**

	*x*	*y*	*z*	*U*_iso_*/*U*_eq_	Occ. (<1)
S1	0.07691 (2)	0.68241 (9)	0.52778 (2)	0.02961 (19)	
O1	0.40973 (6)	0.2937 (3)	0.41323 (7)	0.0373 (5)	
N1	0.43390 (7)	0.3073 (3)	0.50624 (9)	0.0308 (5)	
H1N	0.4631	0.2738	0.4965	0.037*	
N2	0.37906 (7)	0.3899 (3)	0.57444 (8)	0.0299 (5)	
H2	0.3705	0.4104	0.6098	0.036*	
N3	0.35360 (7)	0.3813 (3)	0.47740 (8)	0.0282 (5)	
N4	0.30101 (7)	0.4672 (3)	0.54684 (8)	0.0289 (5)	
N5	0.29244 (7)	0.5038 (3)	0.60491 (8)	0.0300 (5)	
N6	0.17773 (7)	0.5903 (3)	0.53920 (8)	0.0283 (5)	
N7	0.16607 (7)	0.5794 (3)	0.48524 (8)	0.0299 (5)	
N8	0.09905 (7)	0.6256 (3)	0.42446 (9)	0.0332 (5)	
N9	0.03348 (7)	0.7075 (3)	0.47827 (9)	0.0327 (5)	
N10	0.25877 (7)	0.4604 (3)	0.45724 (8)	0.0292 (5)	
H10N	0.2847	0.4226	0.4397	0.035*	
H10M	0.2317	0.4780	0.4377	0.035*	
C1	0.39937 (8)	0.3258 (4)	0.46226 (11)	0.0288 (5)	
C2	0.42627 (9)	0.3368 (4)	0.56318 (11)	0.0308 (6)	
C3	0.34649 (8)	0.4099 (4)	0.53120 (10)	0.0276 (5)	
C4	0.24716 (8)	0.5503 (4)	0.60692 (10)	0.0281 (5)	
C5	0.22433 (8)	0.5469 (4)	0.55086 (10)	0.0285 (6)	
C6	0.26045 (9)	0.4907 (4)	0.51269 (10)	0.0286 (5)	
C7	0.11800 (9)	0.6252 (4)	0.47621 (10)	0.0292 (5)	
C8	0.05135 (9)	0.6725 (4)	0.42785 (11)	0.0331 (6)	
C9	0.02131 (11)	0.6866 (6)	0.37484 (13)	0.0563 (9)	
H9A	−0.0123	0.7067	0.3847	0.084*	
H9B	0.0241	0.5686	0.3529	0.084*	
H9C	0.0324	0.7939	0.3519	0.084*	
C10	0.22345 (9)	0.5893 (4)	0.66330 (11)	0.0321 (6)	
C11	0.26138 (9)	0.5910 (4)	0.71164 (11)	0.0382 (6)	
H11A	0.2841	0.6953	0.7055	0.057*	
H11B	0.2787	0.4696	0.7121	0.057*	
H11C	0.2456	0.6092	0.7482	0.057*	
C12	0.19714 (9)	0.7813 (4)	0.66161 (11)	0.0384 (7)	
H12A	0.1812	0.8021	0.6980	0.058*	
H12B	0.1730	0.7807	0.6305	0.058*	
H12C	0.2203	0.8836	0.6553	0.058*	
C13	0.18754 (9)	0.4262 (5)	0.67302 (11)	0.0412 (7)	
H13A	0.1714	0.4464	0.7092	0.062*	
H13B	0.2048	0.3047	0.6744	0.062*	
H13C	0.1636	0.4239	0.6417	0.062*	
O2	0.45672 (6)	0.3194 (3)	0.60030 (8)	0.0383 (5)	
O4	0.18713 (7)	0.4753 (3)	0.35961 (8)	0.0463 (5)	
H4A	0.1719	0.3757	0.3430	0.056*	
H4B	0.1611	0.5393	0.3707	0.056*	
O3A	0.13169 (17)	0.1331 (6)	0.30823 (16)	0.0335 (13)	0.488 (5)
C14A	0.10177 (11)	0.1786 (4)	0.27041 (11)	0.0290 (15)	0.488 (5)
C15A	0.07874 (15)	0.3737 (4)	0.25815 (16)	0.0457 (16)	0.488 (5)
H15A	0.1031	0.4764	0.2596	0.055*	0.488 (5)
H15B	0.0533	0.4025	0.2857	0.055*	0.488 (5)
C16A	0.05744 (16)	0.3497 (5)	0.19745 (16)	0.0485 (17)	0.488 (5)
H16A	0.0273	0.4232	0.1926	0.058*	0.488 (5)
H16B	0.0805	0.3930	0.1687	0.058*	0.488 (5)
C17A	0.04784 (13)	0.1366 (5)	0.19163 (14)	0.0356 (16)	0.488 (5)
H17A	0.0145	0.1050	0.2021	0.043*	0.488 (5)
H17B	0.0534	0.0923	0.1522	0.043*	0.488 (5)
N11A	0.08266 (10)	0.0533 (4)	0.23192 (11)	0.0322 (12)	0.488 (5)
C18A	0.09354 (17)	−0.1506 (4)	0.23177 (18)	0.0488 (17)	0.488 (5)
H18A	0.1050	−0.1876	0.1941	0.073*	0.488 (5)
H18B	0.0644	−0.2227	0.2403	0.073*	0.488 (5)
H18C	0.1185	−0.1780	0.2608	0.073*	0.488 (5)
O3B	0.12735 (16)	0.2045 (5)	0.31146 (16)	0.0311 (12)	0.512 (5)
C14B	0.10602 (10)	0.1110 (4)	0.27360 (11)	0.0333 (16)	0.512 (5)
C15B	0.10728 (13)	−0.1044 (4)	0.26215 (16)	0.0413 (14)	0.512 (5)
H15C	0.1044	−0.1776	0.2980	0.050*	0.512 (5)
H15D	0.1373	−0.1416	0.2433	0.050*	0.512 (5)
C16B	0.06328 (14)	−0.1355 (4)	0.22232 (17)	0.0423 (14)	0.512 (5)
H16C	0.0695	−0.2378	0.1944	0.051*	0.512 (5)
H16D	0.0347	−0.1699	0.2444	0.051*	0.512 (5)
C17B	0.05587 (13)	0.0551 (5)	0.19218 (13)	0.0394 (16)	0.512 (5)
H17C	0.0727	0.0584	0.1554	0.047*	0.512 (5)
H17D	0.0213	0.0814	0.1851	0.047*	0.512 (5)
N11B	0.07691 (9)	0.1908 (3)	0.23277 (10)	0.0354 (12)	0.512 (5)
C18B	0.06870 (15)	0.3959 (4)	0.22719 (17)	0.0419 (15)	0.512 (5)
H18D	0.0343	0.4226	0.2301	0.063*	0.512 (5)
H18E	0.0799	0.4393	0.1901	0.063*	0.512 (5)
H18F	0.0864	0.4630	0.2577	0.063*	0.512 (5)

### Atomic displacement parameters (Å^2^)

**Table:**

	*U*^11^	*U*^22^	*U*^33^	*U*^12^	*U*^13^	*U*^23^
S1	0.0231 (3)	0.0402 (4)	0.0255 (3)	0.0041 (2)	0.0000 (2)	−0.0017 (3)
O1	0.0262 (9)	0.0595 (13)	0.0264 (9)	0.0060 (8)	0.0017 (7)	−0.0019 (8)
N1	0.0183 (10)	0.0433 (13)	0.0307 (11)	0.0024 (9)	0.0007 (8)	−0.0018 (9)
N2	0.0233 (10)	0.0437 (13)	0.0227 (10)	0.0029 (9)	−0.0004 (8)	−0.0026 (9)
N3	0.0214 (10)	0.0370 (12)	0.0262 (11)	0.0010 (8)	0.0007 (8)	0.0011 (9)
N4	0.0228 (10)	0.0396 (13)	0.0241 (11)	0.0026 (9)	0.0001 (8)	−0.0009 (9)
N5	0.0244 (11)	0.0404 (13)	0.0253 (11)	0.0031 (9)	0.0021 (8)	−0.0026 (9)
N6	0.0259 (10)	0.0317 (12)	0.0271 (11)	0.0010 (8)	−0.0007 (8)	0.0004 (9)
N7	0.0232 (10)	0.0397 (13)	0.0265 (11)	0.0024 (9)	−0.0023 (8)	0.0000 (9)
N8	0.0266 (11)	0.0457 (14)	0.0272 (11)	0.0074 (9)	−0.0014 (9)	−0.0016 (10)
N9	0.0250 (11)	0.0437 (14)	0.0291 (11)	0.0057 (9)	−0.0020 (8)	−0.0013 (9)
N10	0.0243 (10)	0.0402 (13)	0.0231 (10)	0.0054 (9)	−0.0001 (8)	−0.0008 (9)
C1	0.0230 (12)	0.0336 (15)	0.0298 (13)	0.0001 (10)	0.0003 (10)	0.0012 (10)
C2	0.0238 (12)	0.0367 (15)	0.0318 (13)	0.0011 (10)	−0.0006 (10)	−0.0001 (11)
C3	0.0212 (12)	0.0323 (14)	0.0294 (13)	0.0005 (9)	−0.0003 (9)	0.0002 (10)
C4	0.0239 (12)	0.0338 (14)	0.0266 (13)	0.0028 (10)	0.0002 (9)	0.0001 (10)
C5	0.0260 (12)	0.0334 (14)	0.0260 (12)	0.0043 (10)	−0.0009 (10)	−0.0002 (10)
C6	0.0251 (12)	0.0320 (14)	0.0287 (13)	0.0008 (10)	−0.0019 (9)	0.0015 (10)
C7	0.0270 (13)	0.0337 (14)	0.0269 (12)	0.0050 (10)	0.0012 (10)	−0.0010 (10)
C8	0.0286 (13)	0.0426 (16)	0.0279 (13)	0.0079 (11)	−0.0029 (10)	−0.0014 (11)
C9	0.0387 (16)	0.097 (3)	0.0328 (16)	0.0236 (17)	−0.0070 (13)	−0.0088 (16)
C10	0.0233 (12)	0.0473 (17)	0.0258 (13)	0.0029 (11)	0.0035 (10)	−0.0005 (11)
C11	0.0324 (14)	0.0559 (18)	0.0264 (13)	0.0058 (12)	0.0007 (10)	−0.0014 (12)
C12	0.0321 (14)	0.0549 (19)	0.0282 (14)	0.0083 (12)	0.0016 (11)	−0.0045 (12)
C13	0.0325 (15)	0.059 (2)	0.0319 (15)	−0.0042 (13)	0.0063 (11)	0.0026 (13)
O2	0.0251 (9)	0.0573 (13)	0.0323 (10)	0.0053 (8)	−0.0068 (7)	−0.0020 (8)
O4	0.0331 (10)	0.0676 (15)	0.0383 (11)	0.0036 (9)	0.0018 (8)	−0.0084 (10)
O3A	0.030 (2)	0.051 (3)	0.020 (2)	0.018 (2)	−0.0027 (17)	−0.018 (2)
C14A	0.016 (2)	0.051 (3)	0.021 (3)	0.004 (2)	−0.001 (2)	0.001 (2)
C15A	0.040 (3)	0.048 (4)	0.049 (4)	0.008 (3)	0.001 (3)	−0.001 (3)
C16A	0.053 (4)	0.052 (4)	0.040 (3)	0.012 (3)	−0.007 (3)	0.005 (3)
C17A	0.031 (3)	0.044 (4)	0.032 (3)	0.007 (3)	−0.004 (2)	−0.004 (3)
N11A	0.031 (2)	0.041 (3)	0.024 (2)	0.002 (2)	−0.0028 (18)	−0.001 (2)
C18A	0.060 (4)	0.044 (4)	0.042 (4)	0.008 (3)	0.001 (3)	−0.004 (3)
O3B	0.032 (2)	0.030 (3)	0.031 (2)	0.0077 (19)	−0.0010 (18)	−0.0098 (18)
C14B	0.032 (3)	0.039 (3)	0.030 (3)	−0.001 (2)	0.012 (3)	−0.002 (2)
C15B	0.040 (3)	0.041 (3)	0.043 (3)	−0.001 (2)	−0.004 (3)	0.000 (3)
C16B	0.043 (3)	0.043 (3)	0.040 (3)	−0.006 (3)	−0.001 (3)	−0.003 (2)
C17B	0.032 (3)	0.051 (4)	0.035 (3)	0.004 (3)	−0.003 (2)	−0.007 (3)
N11B	0.030 (2)	0.043 (3)	0.032 (2)	−0.0011 (19)	−0.0004 (19)	0.000 (2)
C18B	0.042 (3)	0.043 (3)	0.040 (4)	0.000 (3)	−0.001 (3)	0.002 (3)

### Geometric parameters (Å, °)

**Table:**

S1—N9	1.665 (2)	C12—H12B	0.9800
S1—C7	1.731 (2)	C12—H12C	0.9800
O1—C1	1.213 (3)	C13—H13A	0.9800
N1—C2	1.372 (3)	C13—H13B	0.9800
N1—C1	1.399 (3)	C13—H13C	0.9800
N1—H1N	0.8800	O4—H4A	0.9018
N2—C3	1.351 (3)	O4—H4B	0.8962
N2—C2	1.397 (3)	O3A—C14A	1.2437
N2—H2	0.8800	C14A—N11A	1.3603
N3—C3	1.297 (3)	C14A—C15A	1.5375
N3—C1	1.386 (3)	C15A—C16A	1.5378
N4—C6	1.378 (3)	C15A—H15A	0.9900
N4—C3	1.385 (3)	C15A—H15B	0.9900
N4—N5	1.411 (3)	C16A—C17A	1.5263
N5—C4	1.304 (3)	C16A—H16A	0.9900
N6—N7	1.301 (3)	C16A—H16B	0.9900
N6—C5	1.354 (3)	C17A—N11A	1.4598
N7—C7	1.388 (3)	C17A—H17A	0.9900
N8—C7	1.311 (3)	C17A—H17B	0.9900
N8—C8	1.372 (3)	N11A—C18A	1.4644
N9—C8	1.316 (3)	C18A—H18A	0.9800
N10—C6	1.317 (3)	C18A—H18B	0.9800
N10—H10N	0.8800	C18A—H18C	0.9800
N10—H10M	0.8800	O3B—C14B	1.2436
C2—O2	1.207 (3)	C14B—N11B	1.3603
C4—C5	1.446 (3)	C14B—C15B	1.5375
C4—C10	1.516 (3)	C15B—C16B	1.5379
C5—C6	1.417 (3)	C15B—H15C	0.9900
C8—C9	1.485 (3)	C15B—H15D	0.9900
C9—H9A	0.9800	C16B—C17B	1.5263
C9—H9B	0.9800	C16B—H16C	0.9900
C9—H9C	0.9800	C16B—H16D	0.9900
C10—C11	1.530 (3)	C17B—N11B	1.4599
C10—C12	1.535 (4)	C17B—H17C	0.9900
C10—C13	1.541 (4)	C17B—H17D	0.9900
C11—H11A	0.9800	N11B—C18B	1.4644
C11—H11B	0.9800	C18B—H18D	0.9800
C11—H11C	0.9800	C18B—H18E	0.9800
C12—H12A	0.9800	C18B—H18F	0.9800
			
N9—S1—C7	91.05 (11)	C10—C12—H12C	109.5
C2—N1—C1	125.7 (2)	H12A—C12—H12C	109.5
C2—N1—H1N	117.1	H12B—C12—H12C	109.5
C1—N1—H1N	117.1	C10—C13—H13A	109.5
C3—N2—C2	120.2 (2)	C10—C13—H13B	109.5
C3—N2—H2	119.9	H13A—C13—H13B	109.5
C2—N2—H2	119.9	C10—C13—H13C	109.5
C3—N3—C1	117.1 (2)	H13A—C13—H13C	109.5
C6—N4—C3	128.4 (2)	H13B—C13—H13C	109.5
C6—N4—N5	112.57 (19)	H4A—O4—H4B	98.0
C3—N4—N5	118.97 (19)	O3A—C14A—N11A	123.4
C4—N5—N4	105.43 (19)	O3A—C14A—C15A	129.2
N7—N6—C5	113.5 (2)	N11A—C14A—C15A	107.4
N6—N7—C7	110.6 (2)	C14A—C15A—C16A	103.0
C7—N8—C8	108.4 (2)	C14A—C15A—H15A	111.2
C8—N9—S1	108.82 (17)	C16A—C15A—H15A	111.2
C6—N10—H10N	120.0	C14A—C15A—H15B	111.2
C6—N10—H10M	120.0	C16A—C15A—H15B	111.2
H10N—N10—H10M	120.0	H15A—C15A—H15B	109.1
O1—C1—N3	122.4 (2)	C17A—C16A—C15A	104.7
O1—C1—N1	120.5 (2)	C17A—C16A—H16A	110.8
N3—C1—N1	117.2 (2)	C15A—C16A—H16A	110.8
O2—C2—N1	124.3 (2)	C17A—C16A—H16B	110.8
O2—C2—N2	122.6 (2)	C15A—C16A—H16B	110.8
N1—C2—N2	113.1 (2)	H16A—C16A—H16B	108.9
N3—C3—N2	126.8 (2)	N11A—C17A—C16A	102.9
N3—C3—N4	117.6 (2)	N11A—C17A—H17A	111.2
N2—C3—N4	115.6 (2)	C16A—C17A—H17A	111.2
N5—C4—C5	111.6 (2)	N11A—C17A—H17B	111.2
N5—C4—C10	121.2 (2)	C16A—C17A—H17B	111.2
C5—C4—C10	127.2 (2)	H17A—C17A—H17B	109.1
N6—C5—C6	128.7 (2)	C14A—N11A—C17A	114.4
N6—C5—C4	125.3 (2)	C14A—N11A—C18A	123.9
C6—C5—C4	106.0 (2)	C17A—N11A—C18A	121.7
N10—C6—N4	124.3 (2)	O3B—C14B—N11B	123.4
N10—C6—C5	131.2 (2)	O3B—C14B—C15B	129.2
N4—C6—C5	104.5 (2)	N11B—C14B—C15B	107.4
N8—C7—N7	120.5 (2)	C14B—C15B—C16B	103.0
N8—C7—S1	112.82 (18)	C14B—C15B—H15C	111.2
N7—C7—S1	126.71 (18)	C16B—C15B—H15C	111.2
N9—C8—N8	118.9 (2)	C14B—C15B—H15D	111.2
N9—C8—C9	121.5 (2)	C16B—C15B—H15D	111.2
N8—C8—C9	119.6 (2)	H15C—C15B—H15D	109.1
C8—C9—H9A	109.5	C17B—C16B—C15B	104.7
C8—C9—H9B	109.5	C17B—C16B—H16C	110.8
H9A—C9—H9B	109.5	C15B—C16B—H16C	110.8
C8—C9—H9C	109.5	C17B—C16B—H16D	110.8
H9A—C9—H9C	109.5	C15B—C16B—H16D	110.8
H9B—C9—H9C	109.5	H16C—C16B—H16D	108.9
C4—C10—C11	109.9 (2)	N11B—C17B—C16B	102.9
C4—C10—C12	110.7 (2)	N11B—C17B—H17C	111.2
C11—C10—C12	109.4 (2)	C16B—C17B—H17C	111.2
C4—C10—C13	107.0 (2)	N11B—C17B—H17D	111.2
C11—C10—C13	109.6 (2)	C16B—C17B—H17D	111.2
C12—C10—C13	110.3 (2)	H17C—C17B—H17D	109.1
C10—C11—H11A	109.5	C14B—N11B—C17B	114.4
C10—C11—H11B	109.5	C14B—N11B—C18B	123.9
H11A—C11—H11B	109.5	C17B—N11B—C18B	121.7
C10—C11—H11C	109.5	N11B—C18B—H18D	109.5
H11A—C11—H11C	109.5	N11B—C18B—H18E	109.5
H11B—C11—H11C	109.5	H18D—C18B—H18E	109.5
C10—C12—H12A	109.5	N11B—C18B—H18F	109.5
C10—C12—H12B	109.5	H18D—C18B—H18F	109.5
H12A—C12—H12B	109.5	H18E—C18B—H18F	109.5
			
C6—N4—N5—C4	−0.2 (3)	C8—N8—C7—N7	179.1 (2)
C3—N4—N5—C4	−178.7 (2)	C8—N8—C7—S1	0.0 (3)
C5—N6—N7—C7	180.0 (2)	N6—N7—C7—N8	179.1 (2)
C7—S1—N9—C8	0.2 (2)	N6—N7—C7—S1	−2.0 (3)
C3—N3—C1—O1	179.3 (3)	N9—S1—C7—N8	−0.1 (2)
C3—N3—C1—N1	−0.6 (3)	N9—S1—C7—N7	−179.1 (2)
C2—N1—C1—O1	−178.9 (3)	S1—N9—C8—N8	−0.2 (3)
C2—N1—C1—N3	0.9 (4)	S1—N9—C8—C9	−179.0 (2)
C1—N1—C2—O2	179.8 (2)	C7—N8—C8—N9	0.1 (4)
C1—N1—C2—N2	−0.2 (4)	C7—N8—C8—C9	178.9 (3)
C3—N2—C2—O2	179.1 (2)	N5—C4—C10—C11	7.4 (4)
C3—N2—C2—N1	−0.9 (3)	C5—C4—C10—C11	−176.5 (3)
C1—N3—C3—N2	−0.5 (4)	N5—C4—C10—C12	128.4 (3)
C1—N3—C3—N4	179.4 (2)	C5—C4—C10—C12	−55.6 (3)
C2—N2—C3—N3	1.3 (4)	N5—C4—C10—C13	−111.4 (3)
C2—N2—C3—N4	−178.6 (2)	C5—C4—C10—C13	64.6 (3)
C6—N4—C3—N3	3.2 (4)	O3A—C14A—C15A—C16A	164.1
N5—N4—C3—N3	−178.5 (2)	N11A—C14A—C15A—C16A	−17.2
C6—N4—C3—N2	−176.8 (2)	C14A—C15A—C16A—C17A	26.5
N5—N4—C3—N2	1.4 (3)	C15A—C16A—C17A—N11A	−26.1
N4—N5—C4—C5	−0.2 (3)	O3A—C14A—N11A—C17A	179.4
N4—N5—C4—C10	176.4 (2)	C15A—C14A—N11A—C17A	0.6
N7—N6—C5—C6	−0.9 (4)	O3A—C14A—N11A—C18A	2.4
N7—N6—C5—C4	179.0 (2)	C15A—C14A—N11A—C18A	−176.4
N5—C4—C5—N6	−179.3 (2)	C16A—C17A—N11A—C14A	16.4
C10—C4—C5—N6	4.3 (4)	C16A—C17A—N11A—C18A	−166.5
N5—C4—C5—C6	0.5 (3)	O3B—C14B—C15B—C16B	164.0
C10—C4—C5—C6	−175.8 (2)	N11B—C14B—C15B—C16B	−17.2
C3—N4—C6—N10	0.1 (4)	C14B—C15B—C16B—C17B	26.5
N5—N4—C6—N10	−178.2 (2)	C15B—C16B—C17B—N11B	−26.1
C3—N4—C6—C5	178.9 (2)	O3B—C14B—N11B—C17B	179.4
N5—N4—C6—C5	0.5 (3)	C15B—C14B—N11B—C17B	0.6
N6—C5—C6—N10	−2.2 (5)	O3B—C14B—N11B—C18B	2.4
C4—C5—C6—N10	178.0 (3)	C15B—C14B—N11B—C18B	−176.4
N6—C5—C6—N4	179.2 (3)	C16B—C17B—N11B—C14B	16.4
C4—C5—C6—N4	−0.6 (3)	C16B—C17B—N11B—C18B	−166.5

### Hydrogen-bond geometry (Å, °)

**Table:**

*D*—H···*A*	*D*—H	H···*A*	*D*···*A*	*D*—H···*A*
O4—H4A···O3A	0.902	2.186	3.087 (4)	175.8
O4—H4A···O3B	0.902	1.868	2.752 (4)	166.3
O4—H4B···N7	0.896	2.700	3.104 (2)	108.5
O4—H4B···N8	0.896	2.245	3.100 (2)	159.4
N1—H1N···N9^i^	0.88	2.07	2.947 (2)	176
N2—H2···N5	0.88	2.27	2.654 (2)	106
N2—H2···O3A^ii^	0.88	1.95	2.778 (4)	157
N2—H2···O3B^ii^	0.88	2.01	2.766 (4)	143
N10—H10N···N3	0.88	2.11	2.727 (2)	126
N10—H10M···O4	0.88	2.19	3.002 (2)	154
N10—H10M···N7	0.88	2.28	2.804 (2)	119

Symmetry codes: (i) *x*+1/2, *y*−1/2, *z*; (ii) −*x*+1/2, −*y*+1/2, −*z*+1.
